# Antisocial personality disorder in women: a cross-sectional study about 20 cases

**DOI:** 10.1192/j.eurpsy.2022.1720

**Published:** 2022-09-01

**Authors:** M. Karoui, S. Khanfir, G. Amri, R. Kammoun, H. Nefzi, F. Ellouz

**Affiliations:** 1 Razi Hospital, Psychiatry G, manouba, Tunisia; 2 Razi Hospital, Manouba, tunis, Tunisia; 3 Razi hospital, Psychiatry G, manouba, Tunisia; 4 Razi hospital, Psychiatry G Department, Mannouba, Tunisia; 5 Razi hospital, Psychiatry G, denden, Tunisia

**Keywords:** violence, Antisocial Personality disorder, women

## Abstract

**Introduction:**

Antisocial personality disorder (ASPD) is characterized by a long term pattern of disregard for, or violation of the rights of others that occurs in childhood or early adolescence and continues into adulthood. This disorder remains unknown in women.

**Objectives:**

The aim of this study was to describe socio-demographic, clinical, psychometric and therapeutic characteristics of women with ASPD hospitalized in psychiatric ward.

**Methods:**

A cross-sectional and descriptive study was conducted in G psychiatry department, in Razi hospital. It included 20 women with ASPD and hospitalized in psychiatric ward. Socio-demographic, clinical and therapeutic characteristics were assessed. A psychometric evaluation was carried out by the application of six scales: BIS 11, HCR 20, VRAG, PCL-R, ENFP and PID5-BF.

**Results:**

The mean age of the patients was 34 ± 9 years. Patients with a personal history of suicide attempt accounted for 45% of the study population. Patients with a criminal history accounted for 67.5% of the study population. A substance-related disorder was found in 85% of the patients. Adjustment Disorder was retained in 42.5% of the patients and Psychotic Disorder was diagnosed in 32.5% of the population. The average score at VRAG was 6.18 ± 5.8. The mean score at PCL R was 24 ± 4.2. High impulsivity scores were found.

**Conclusions:**

ASPD represents a major concern for clinicians in psychiatric wards. A better knowledge of the characteristics of this trouble in women could improve their quality of care.

**Disclosure:**

No significant relationships.

